# High Stressor Exposure and Low Stressor Diversity Are Linked to Higher Blood Pressure Across Age

**DOI:** 10.1093/geroni/igaa057.2251

**Published:** 2020-12-16

**Authors:** Rachel Koffer, Kristina Dickman, Thomas Kamarck

**Affiliations:** University of Pittsburgh, Pittsburgh, Pennsylvania, United States

## Abstract

Stress exposure is linked to elevated blood pressure, which increases risk for cardiovascular disease (Spruill, 2010; WHO, 2013). Stress exposure may be especially harmful when concentrated in one particular domain (i.e., low stressor diversity) (Koffer, et al., 2016). Using a diversity index, we test whether high stressor exposure and low stressor diversity is associated with high resting blood pressure. Participants (N=391, aged 40-64 years) completed four days of hourly self-report of stressful experiences (e.g., work task demand, non-work task demand, arguments, interpersonal tension), with clinic blood pressure separately assessed. Linear regression results indicate older adults experienced lower stressor diversity (B = -0.003, p =.003). Further, higher stressor exposure with lower stressor diversity related to higher diastolic blood pressure (B= -7.21, p=.046). Experiencing high stress concentrated in one domain may increase risk of high blood pressure. We discuss how low stressor diversity may help explain age-related risk of cardiovascular disease.

